# Cytokine profile in severe gram-positive and gram-negative abdominal sepsis

**DOI:** 10.1038/srep11355

**Published:** 2015-06-16

**Authors:** Maja Surbatovic, Nada Popovic, Danilo Vojvodic, Ivan Milosevic, Gordana Acimovic, Milan Stojicic, Milic Veljovic, Jasna Jevdjic, Dragan Djordjevic, Sonja Radakovic

**Affiliations:** 1 Clinic of Anesthesiology and Intensive Therapy, Military Medical Academy, Crnotravska 17, Belgrade 11000, Serbia; 2 Faculty of Medicine of the Military Medical Academy, University of Defence, Belgrade, Crnotravska 17, Belgrade 11000, Serbia; 3 School of Medicine, University of Belgrade, Dr Subotica 8, Belgrade 11000, Serbia; 4 Institute for Medical Research, Military Medical Academy, Crnotravska 17, Belgrade 11000, Serbia; 5 Clinical Center of Serbia, Pasterova 2, Belgrade 11000, Serbia; 6 Clinical Center Kragujevac, Zmaj Jovina 30, Kragujevac 34000, Serbia; 7 Faculty of Medical Sciences University of Kragujevac, Svetozara Markovica 69,Kragujevac 34000, Serbia; 8 Sector of Preventive Medicine, Military Medical Academy, Crnotravska 17, Belgrade 11000, Serbia

## Abstract

Sepsis is a principal cause of death in critical care units worldwide and consumes considerable healthcare resources. The aim of our study was to determine whether the early cytokine profile can discriminate between Gram-positive and Gram-negative bacteraemia (GPB and GNB, respectively) and to assess the prognostic value regarding outcome in critically ill patients with severe abdominal sepsis. The outcome measure was hospital mortality. Blood samples were obtained from 165 adult patients with confirmed severe abdominal sepsis. Levels of the proinflammatory mediators TNF-α, IL-8, IL-12 and IFN-γ and the anti-inflammatory mediators IL-1ra, IL-4, IL-10 and TGF-β1 were determined and correlated with the nature of the bacteria isolated from the blood culture and outcome. The cytokine profile in our study indicated that the TNF-α levels were 2-fold, IL-8 were 3.3-fold, IFN-γ were 13-fold, IL-1ra were 1.05-fold, IL-4 were 1.4-fold and IL-10 were 1.83-fold higher in the GNB group compared with the GPB group. The TNF-α levels were 4.7-fold, IL-8 were 4.6-fold, IL-1ra were 1.5-fold and IL-10 were 3.3-fold higher in the non-survivors compared with the survivors.

Sepsis is a principal cause of death in critical care units worldwide and consumes considerable healthcare resources. The average annual increase in the incidence of severe sepsis is approximately 13%^1^. The immuno-inflammatory response in critically ill septic patients is very complex^2^. Despite emerging fundamental differences in the host immune response to Gram-positive bacterial pathogens compared with Gram-negative microorganisms, current clinical dogma dictates that both pathogens should be treated with similar therapeutic protocols. However, evidence suggests that there are different mechanisms of the clinical manifestations of Gram-positive and Gram-negative sepsis to the extent that they may represent different disease entities^3^. A host may respond differently to lipopolysaccharide (LPS) of Gram-negative bacteria and lipoteichoic acid (LTA) of Gram-positive bacteria. Some microbial challenges may elicit levels of mediators that damage both the infecting microorganism and the host. Furthermore, Gram-positive and Gram-negative bacteria may induce different inflammatory patterns^4^.

Cytokines comprise a group of endogenous inflammatory mediators and immunomodulatory proteins that are key mediators in sepsis. They are broadly divided into proinflammatory and anti-inflammatory mediators. We have chosen four proinflammatory mediators, including tumour necrosis factor (TNF)-α, interleukin (IL)-8, IL-12, and interferon (IFN)-γ, and four anti-inflammatory mediators, including interleukin 1 receptor antagonist (IL-1ra), IL-4, IL-10 and transforming growth factor (TGF)-β1, to assess the cytokine profile in Gram-positive and Gram-negative severe sepsis.

The aim of our study was to determine whether the early cytokine profile can discriminate between Gram-positive and Gram-negative bacteraemia in patients with severe abdominal sepsis and to assess the prognostic value regarding outcome in this patient population. The outcome measure was hospital mortality.

## Results

One hundred sixty-five patients (average age was 52.5 years; range: 19–81 years; 104 females, 61 males) with severe peritonitis as the underlying cause of severe secondary sepsis or septic shock were enrolled. Of the 165 patients, 80 patients (48.5%) developed Gram-positive bacteraemia-GPB, 65 patients (39.4%) developed Gram-negative—GNB, and 20 patients (12.1%) had polymicrobial bacteraemia-POLY. The demographic data are shown on Table 1. A comparison of the GPB, GNB and POLY groups according to outcome indicated there was a significant difference in the mortality rates. In GPB group mortality was 43.8%, in GNB group 63.1% and in POLY group 60.0%; χ^2^ was 5.789 with exact p value of 0.045. Further analysis demonstrated that the difference between GPB and GNB was significant (Table 2) with the highest mortality in the GNB group. The mortality rates were similar in the GNB and POLY groups. The difference in the mortality rates between the GPB and POLY groups was not significant most likely because of the relatively small number of patients in the POLY group.

The most frequent Gram-positive bacteria in our study were Coagulase-negative staphylococci, Staphylococcus aureus and Enterococci; less frequent Gram-positive pathogens were Streptococcus and Bacillaceae. The most frequent Gram-negative bacteria were Pseudomonas aeruginosa, Acinetobacter baumannii and Klebsiella pneumoniae; less frequent Gram-negative pathogens were Escherichia coli, Proteus vulgaris and Citrobacter species.

### Inflammatory mediators and nature of bacteraemia

The median values and comparisons according to the nature of the bacteraemia are shown in Table 3. When the three groups of patients (GPB, GNB, and POLY) were compared, we identified significant differences in the TNF-α, IL-8, IFN-γ, IL-1ra, IL-4 and IL-10 values.

The TNF-α levels were 2-fold higher in the GNB group and 1.7-fold higher in the POLY group compared with the GPB group. The IL-8 levels were 3.3-fold higher in the GNB group and 13-fold higher in the POLY group compared with the GPB group; the IL-8 levels were also 3.9-fold higher in the POLY group compared with the GNB group. The IFN-γ levels were 13-fold higher in the GNB group compared with the GPB group and 8.6-fold higher in the POLY group compared with the GNB group. The IL-1ra levels were 2.1-fold higher in the POLY group compared with the GPB group. The IL-4 levels were 1.4-fold higher in the GNB group compared with either the GPB or POLY groups. The IL-10 levels were 1.83-fold higher in the GNB group and 1.75-fold higher in the POLY group compared with the GPB group. There was no significant difference in the IL-12 and TGF-β1 values between these three groups.

**Figure 1:**
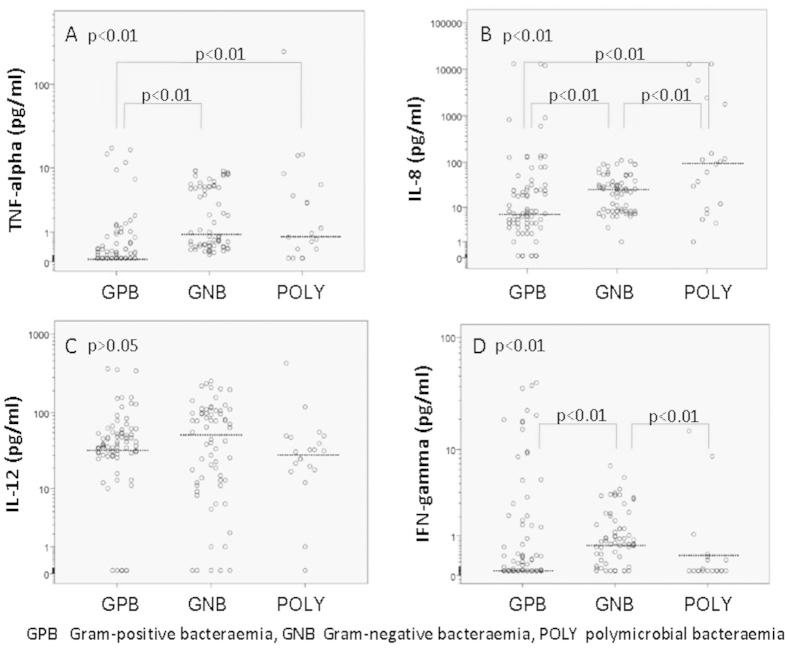
Serum proinflammatory cytokine TNF-α, IL-8, IL-12 and IFN-γ levels according to the nature of the bacteraemia.

**Figure 2:**
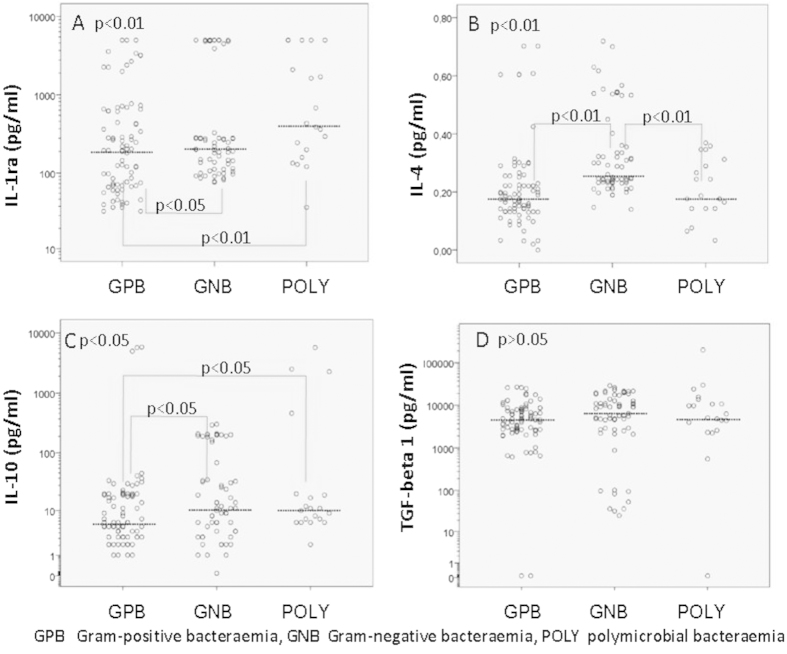
Serum anti-inflammatory cytokine IL-1ra, IL-4, IL-10 and TGF-β1 levels according to the nature of the bacteraemia.

The serum cytokine levels according to the nature of the bacteraemia (GPB, GNB, and POLY) are shown in Figs 1,2 (thick horizontal lines represent the median levels). Because of the wide distribution of serum cytokine concentrations in all figures with the exception of Fig. 2B, logarithm (log 10) transformation of the y axis was performed. This transformation provides more apparent insight in the obtained cytokine values regarding patient categories.

**Figure 3:**
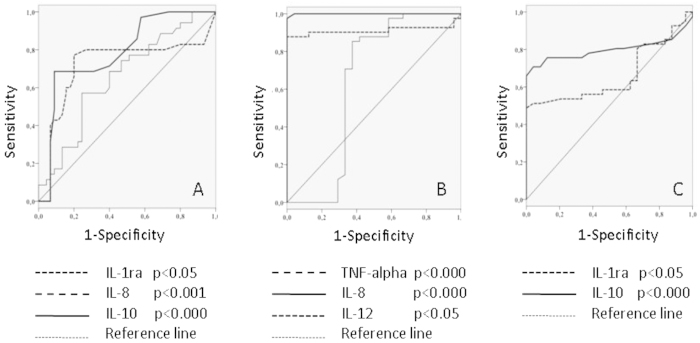
Area under the ROC curve for IL-1ra, IL-8 and IL-10 individual cytokine measurements and the presence of Gram-positive abdominal sepsis (3A); TNF-α, IL-8, IL-12 (3B) and IL-1ra, IL-10 (3C) measurements and Gram-negative abdominal sepsis.

Receiver operator curves were generated to determine the cut-off values for optimal sensitivity and specificity for the IL-1ra, IL-8 and IL-10 levels for Gram-positive bacteraemia. They are all fairly good predictors of Gram-positive sepsis in our patient population (Fig. 3A, Table 4). The patients with values lower than these cut-off levels had a higher probability of developing Gram-positive sepsis compared with the patients with higher values. The area under the curve (AUC) for TNF-α, IL-4, IFN-γ, IL-12 and TGF-β1 was <0.55; thus, these inflammatory mediators failed to predict Gram-positive sepsis in our patient population.

Receiver operator curves were generated to determine the cut-off values for optimal sensitivity and specificity for the TNF-α, IL-8, IL-12, IL-1ra and IL-10 levels for Gram-negative bacteraemia. They are all fairly good predictors of Gram-negative sepsis in our patient population (Fig. 3B,C; Table 4). The patients with higher values than these cut-off levels had a higher probability of developing Gram-negative sepsis compared with the patients with lower values. The area under the curve (AUC) for IL-4, IFN-γ and TGF-β1 was <0.55; thus, these inflammatory mediators failed to predict Gram-negative sepsis in our patient population.

### Correlation between inflammatory mediators

A Spearman rho test of correlation between the inflammatory mediators was performed. In general, there were significantly positive correlations between the proinflammatory mediators TNF-α, IL-8, IL-12 and IFN-γ (Table 5). The only exception was the correlation between IL-8 and IFN-γ, which was not significant.

Regarding the anti-inflammatory mediators, there was a significantly positive correlation between IL-1ra and IL-10 (Table 5). There were also significantly negative correlations between TGF-β1 and IL-1ra or IL-10; there was a significantly positive correlation between TGF-β1 and IL-4. The correlation between IL-10 and IL-4 was not significant.

### Inflammatory mediators and outcome

The median values and comparison of inflammatory mediators according to outcome are shown in Table 6. The overall hospital mortality was 53.3%.

When we compared the non-survivors and survivors, we identified a significant difference (p < 0.01) in the values of TNF-α (4.7-fold higher in the non-survivors), IL-8 (4.6-fold higher in the non-survivors), IL-1ra (1.5-fold higher in the non-survivors), IL-10 (3.3-fold higher in the non-survivors) and TGF-β1 (2-fold higher in the survivors), as well as the IL-4 values (p < 0.05), which were higher in the non-survivors. There was no significant difference in the IL-12 or IFN-γ values between the survivors and non-survivors.

**Figure 4:**
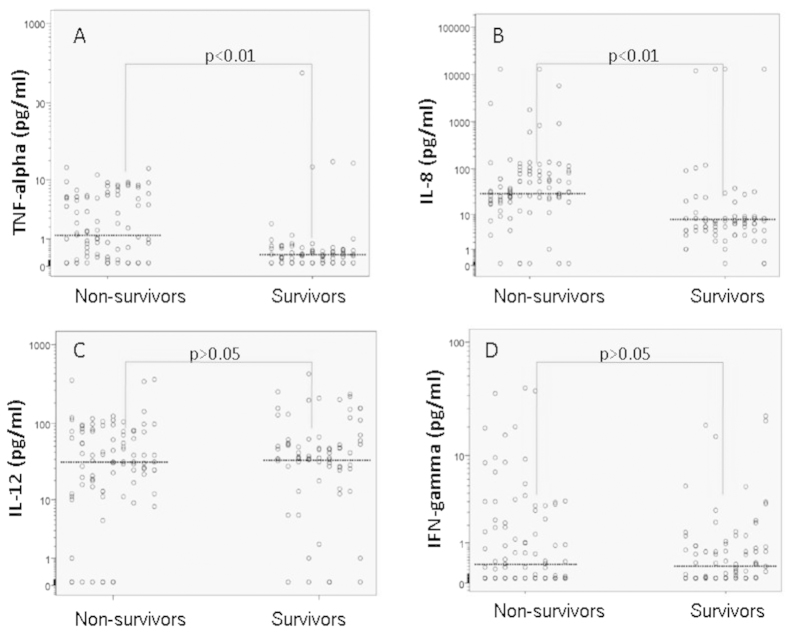
Serum proinflammatory cytokine TNF-α, IL-8, IL-12 and IFN-γ levels according to the outcome.

**Figure 5:**
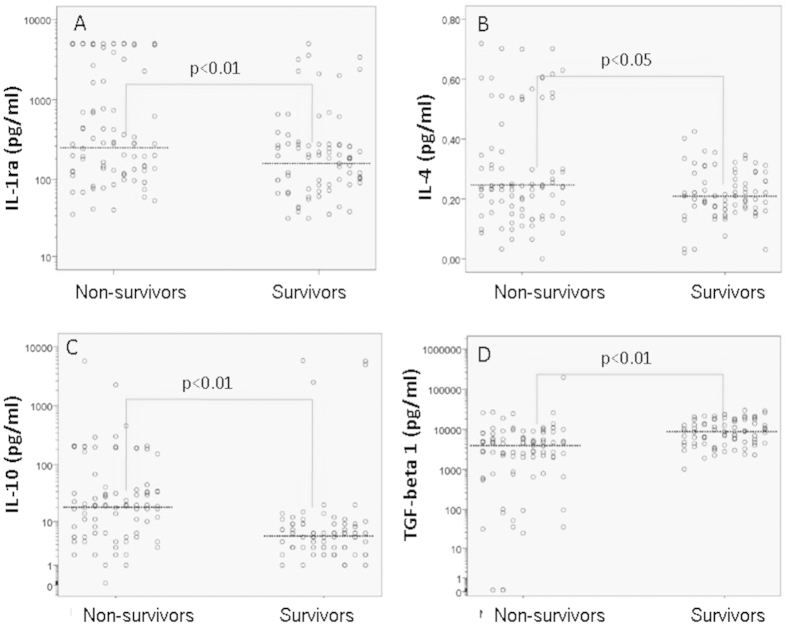
Serum anti-inflammatory cytokine IL-1ra, IL-4, IL-10 and TGF-β1 levels according to the outcome.

The serum cytokine levels according to outcome are shown in Figs 4 and 5 (the thick horizontal lines represent the median levels). Because of the wide distribution of the serum cytokine concentrations in all figures with the exception of Fig. 5B, logarithm (log 10) transformation of the y axis was performed.

**Figure 6:**
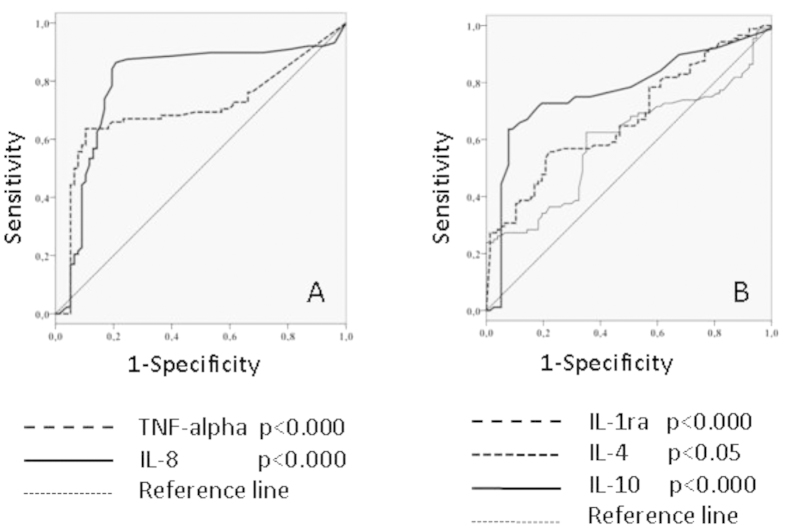
Area under the ROC curve for TNF-α, IL-8 (6A) and IL-1ra, IL-4, IL-10 (6B) measurements and the outcome.

Receiver operator curves were generated to determine the cut-off values for the optimal sensitivity and specificity for the TNF-α, IL-8, IL-1ra, IL-4 and IL-10 levels regarding outcome. These variables are all fairly good predictors of outcome in our patient population (Fig. 6; Table 7). The patients with higher values than the cut-off levels had a higher probability of death compared with the patients with lower values. The area under the curve (AUC) for IFN-γ, IL-12 and TGF-β1 was <0.55; thus, these inflammatory mediators failed to predict outcome.

## Discussion

Microbial infection triggers complex interactions between pathogen and host via host recognition of pathogen-associated molecular patterns (PAMPs). Toll-like receptors (TLRs) are dedicated to the recognition of various bacterial components^5^. For example, LPS is primarily sensed by TLR-4, whereas LTA is sensed by TLR-2. Recognition of PAMPs by TLRs induces inflammatory mediator production^6^. Nevertheless, international guidelines regarding severe sepsis and septic shock treatment do not take into account the type of the causative pathogen^7^. To further complicate this issue, cytokine profile differences are not physiologically or clinically apparent. Signs of systemic inflammatory response syndrome and routine laboratory markers of infection are nonspecific.

Microcirculation is differentially affected by Gram-positive and Gram-negative pathogens^8^. Buerke and co-authors reported that locally administered LPS from *Escherichia coli* enhanced leukocyte-endothelial interaction as opposed to LTA from *Staphylococcus aureus.* However, if live *S. aureus* is systemically applied, it will produce a LPS-like response in part because of peptidoglycan, which is present in both Gram-positive and Gram-negative bacteria; however, Gram-positives in general have a much thicker peptidoglycan layer than Gram-negatives^9^. Additionally, unlike TLR-2 (essential for LTA recognition), TLR-4 (essential for LPS recognition) is widely present on the endothelium^10^. The Gram-positive pathogen *Streptococcus pneumoniae* can cause endothelial damage via exotoxin pneumolysin, which can form pores to enable transfer of pneumococci^11^. The secretion of pro- and anti-inflammatory cytokines occurs in a simultaneous manner from the very first moments in severe sepsis, which form part of an early and concomitant immuno-inflammatory response^12^.

Bjerre and co-authors analysed plasma IFN-γ and IL-10 concentrations in patients with systemic meningococcal disease (Gram-negative) and patients with Gram-positive septic shock caused by *S. pneumoniae* or *S. aureus*. Heat-killed *N. meningitidis*, *S. pneumoniae*, and *S. aureus* boosted cytokine production in a whole blood model^13^. In contrast with our results, the median values of IFN-γ and IL-10 were lower in Gram-negative meningococcal sepsis. In a whole blood model, they demonstrated that *N. meningitidis* induced more IL-10 but less IFN-γ compared with *S. pneumoniae*. This finding is partially in accordance with our results, which indicated that Gram-negative bacteria induce more IL-10 and IFN-γ compared with Gram-positive pathogens. In an experimental study with UV light-killed Gram-positive and Gram-negative bacteria that examined the ability to stimulate monocytes to produce cytokines, the authors demonstrated that in contrast with our results, more TNF-α was released with Gram-positive bacteria compared with Gram-negative bacteria; in accordance with our results, Gram-negative pathogens induced more IL-8^14^ and IL-10^15^ compared with Gram-positive bacteria. Additionally, the ability of heat-killed *E. coli* to induce production of higher concentrations of TNF-α and IL-8 compared with Gram-positive bacteria in neonatal cord blood was reported, similar to our results^16^. In our study, Gram-negative sepsis resulted in a higher plasma concentration of TNF-α than Gram-positive infection. Similar results were reported by other authors, which compared the levels of cytokines in patients with sepsis of differing origins; TNF-α and IL-6 levels were considerably greater in GNB patients^17^. Tang and co-authors evaluated the ability of cytokines to discriminate Gram-positive from Gram-negative sepsis in children with haematology/oncology diseases^18^. They demonstrated that the median levels of TNF-α and IL-10 were significantly higher in Gram-negative bacteraemia, which was similar to our study. The same group recently expanded their investigation^19^. They demonstrated that IL-10 was a good predictor of GNB with a sensitivity and specificity of 70.8 and 80.0%, respectively, with a cut-off level of 50 pg/mL. The TNF-α at a cut-off level of 5 pg/mL had a rather low sensitivity of 54.2% and a very good specificity of 90%. In our study, IL-10, IL-8 and TNF-α were also fairly good predictors of GNB. In contrast with our results, they did not identify a significant difference in the IL-4 or IFN-γ levels between the GPB and GNB groups. Another experimental study^20^ that investigated cytokine concentrations in plasma and peritoneal lavage fluid, after intraperitoneal injection of LTA or LPS, demonstrated that the concentrations of TNF-α, IFN-γ and IL-10 in the plasma significantly increased 1 hour after LPS, but not after LTA. This finding concurs with our findings. Raynor and co-authors examined whether the measurement of plasma cytokines at the time of suspected sepsis could identify patients with bacteraemia in a neonatal intensive care unit^21^. They demonstrated that TNF-α, IL-8, IL-1ra and IL-10 were higher in Gram-negative bacteraemia in accordance with our results. In another study, investigators determined the proinflammatory cytokine profiles of 52 patients with sepsis that resulted from Gram-positive and Gram-negative bacteria^22^. In contrast with our results, they reported similar plasma levels of TNF-α, IL-1ra, IL-8 and IL-10. A group of Japanese authors investigated the effect of Gram-positive pathogens (superantigen Staphylococcal enterotoxin B-SEB) and Gram-negative pathogens (LPS as the outer surface major component) on cytokine release from peripheral blood mononuclear cells (PBMCs)^23^. Similar to our results, the concentrations of TNF-α, IL-1β, IL-6, IL-1ra, IL-10 and sTNF-R2 were much higher as a result of LPS induction. By contrast, SEB had no effect on cytokine release.

The kinetics of cytokine production, not only the pattern, can be different in Gram-positive and Gram-negative infections. Other evidence of two distinct cytokine profiles and kinetics originated from an investigation of S. aureus enterotoxin A and LPS at the cellular level. This evidence can, at least in part, account for the more rapid clinical onset of Gram-negative *E. coli* infection compared with Gram-positive *S. aureus* infection^24^.

Cytokines can influence the activities of other cytokines; thus, we investigated their mutual relations. In general, there were significantly positive correlations between the proinflammatory mediators TNF-α, IL-8, IL-12 and IFN-γ. Skovbjerg and colleagues also identified strong positive correlations between IL-12 and IFN-, TNF-α and IFN-γ, and IL-6 and IL-8, as well as IL-6 and IL-10^25^. The same group of authors have reported that IL-12 production is induced by intact Gram-positive bacteria, but not isolated peptidoglycan or LTA^26^.

Mortality can be influenced by the type of the infecting pathogen. Some investigators have reported that there is increased mortality in staphylococcal infections, in contrast with our results. In another analysis, Gram-negative infections, especially pseudomonal, were associated with a significantly higher mortality rate^27^, which is consistent with our findings. Zahar and colleagues showed no association of the bacteria with mortality^28^. However, other authors find this conclusion to be somewhat misleading because substantial evidence indicates that the characteristics of bacteria influence the clinical presentation and outcome. Also, there is significant proportion of microbiologically undocumented infections^29^.

The identification of septic patients at a higher risk of death is important^30^. Andaluz-Ojeda and colleagues conducted a pilot study^31^ that profiled 17 immune mediators (TNF-α, IL-1β, IL-2, IL-4, IL-5, IL-6, IL-7, IL-8, IL-10, IL-12, IL-13, IL-17, G-CSF, GM-CSF, IFN-γ, MCP-1 and MIP-1β) in the plasma of 29 consecutively recruited septic patients admitted to the ICU. The mortality rate was 41.3%. The IL-6, IL-8, IL-10 and MCP-1 plasma levels were higher in non-survivors, with the most pronounced difference identified in IL-10, which is in accordance with our results.

In severe sepsis and septic shock, IL-10 and TNF-α have been reported to distinguish between survivors and non-survivors at 28 days. The TNF-α and IL-10 levels were higher at the early stages (at admission and the 24th hour) in patients who died. Both cytokines were useful in the prediction of cases that were likely to have a fatal outcome^32^,^33^. In contrast with our results, Bjerre demonstrated that increased IFN-γ concentrations were associated with case fatality^13^. In our previous study, we demonstrated that in patients with severe acute pancreatitis, higher TNF-α levels were associated with better survival^34^, which is different from the patient population in our current study. It is evident that our previous and current results regarding TNF-α point in different directions. Cytokine levels tend to vary considerably. Patients with similar insults can exhibit inter-individual variations, which, at least in part, can be genetically determined^35^,^36^.

Our study has several limitations. First, our patient population was limited to severe abdominal sepsis as a result of peritonitis. We examined as homogenous of a group as possible to minimise other influences on cytokine production, namely, surgical procedures and mechanical ventilation. None of our patients received corticosteroids. However, the general applicability of our results to other forms of sepsis is unclear. Larger studies with different subpopulations of septic patients are warranted. Second, three of eight measured cytokines (TNF-α, IFN-γ and IL-4) had medians below 1 pg/mL. Although the stated sensitivity of the ELISA tests used was below 3 pg/mL according to the manufacturers, the values from our standard curves allowed us to detect concentrations as low as 0.5 pg/mL. Thus, this is an explanation for the inclusion of a number of sample values that were close to the test detection limits. Third, cytokines were only measured at the onset of disease; they were not continuously monitored at different time-points. Thus, the peak levels of these cytokines were unclear. However, as all blood samples were obtained for cytokine analysis immediately after admission to the surgical ICU, we believe there was no significant bias because the timing of the sample collections was similar. Fourth, the microbiological pattern that suggests E. coli is a less frequent causative pathogen observed in our study is different from the majority of the literature. The identification of Coagulase-negative staphylococci (CNS) as one of the most frequent pathogens is not uncommon. Other authors have reported similar findings^19^.

According to guidelines, broad spectrum antibiotics are administered as soon as possible. The lack of causative pathogen identification in more than 40% of cases will make it more difficult to select appropriate antibiotics and may have deleterious effects on the survival of critically ill septic patients^37^,^38^,^39^. Additionally, antimicrobial agents have adverse effects, which may be more pronounced in this patient population^40^.

Using new, rapid and time-effective techniques comparable to ELISA, it could be useful to determine the cytokine profile in critically ill septic patients. In our limited patient population with abdominal sepsis, several cytokines discriminated between Gram-positive and Gram-negative infections and predicted outcome. However, these findings represent preliminary results, and further studies with larger sample sizes and other subpopulations of septic patients are warranted.

## Methods

### Patients

This prospective observational study was approved by the local ethics committee of the Military Medical Academy, and informed consent was obtained from the patient or a first-degree relative. The study was conducted in accordance with the approved guidelines. Blood samples were obtained from 165 adult patients admitted to the surgical ICU with presumed severe sepsis or septic shock, which was subsequently confirmed via blood cultures. The study lasted 3 years and 10 months, and the follow-up period was up to 1 year. The levels of the proinflammatory mediators TNF-α, IL-8, IL-12 and IFN-γ and the anti-inflammatory mediators IL-1ra, IL-4, IL-10 and TGF-β1 were determined and correlated with the nature of bacteria isolated from the blood culture and outcome. Any patient 18 years of age or older who had fulfilled current diagnostic criteria for severe sepsis (sepsis-induced tissue hypoperfusion or organ dysfunction) and septic shock^41^ (for example, requirement for vasoactive support for sepsis-induced hypotension despite adequate fluid resuscitation) was eligible for the study. The diagnostic criteria encompass any of the following variables thought to be a result of the infection: sepsis-induced hypotension, lactate levels greater than 2 mmol/L, urine output less than 0.5 mL/kg/hr for more than two hours despite adequate fluid resuscitation, acute lung injury with PaO_2_/FiO_2_ less than 250, creatinine greater than 2.0 mg/dL (176.8 micromol/L), bilirubin greater than 2.0 mg/dL (34.2 micromol/L), platelet count less than 100,000 and coagulopathy (international normalised ratio—INR) greater than 1.5. The exclusion criteria were as follows: severe sepsis with an underlying cause other than severe peritonitis; previous surgical procedures during ongoing hospitalisation; or microbiologically undocumented severe sepsis. Eighty-five patients were excluded out of 250 patients initially considered for enrolment.

The blood sample for cytokine measurement was drawn immediately after admission to the surgical ICU of the Military Medical Academy (academic tertiary centre) when severe sepsis or septic shock was diagnosed. Blood was simultaneously drawn for a blood culture. Blood sampling was performed according to guidelines: two sets from different sites were percutaneously drawn prior to the administration of antimicrobial therapy in the ICU. Only patients with the same microorganisms isolated from both sites were enrolled.

The Sequential Organ Failure Assessment (SOFA) score^42^, the Simplified Acute Physiology Score (SAPS) II^43^ and the Acute Physiology and Chronic Health Evaluation (APACHE) II score^44^ were calculated and recorded within the first 24 hours after admission to the ICU.

The use of antibiotics, circulatory volume replacement and vasoactive support were performed according to guidelines^41^. Various modes of mechanical ventilation and surgical procedures were performed if and when necessary in all patients.

### Cytokine profiling

A single blood sample was obtained from each patient in tubes that contained ethylenediaminetetraacetic acid (EDTA) immediately after admission to the surgical ICU. Plasma samples were obtained after proper centrifugation and stored at −80 °C until cytokine profiling. The cytokines were measured in plasma using commercial ELISA tests (for IL-4, IL-12 and IFN-γ, Diaclone Research, France; for IL-1ra, Quantikine R&D Systems, USA; for TNF-α, IL-10 and IL-8, Milenia Biotec, Bad Nauheim, Germany; and for TGF-β1, EIA France), following the manufacturers’ instructions and protocols. The cytokine concentration is displayed in pg/mL.

### Statistical analysis

All variables were tested for normal distribution (Kolmogorov-Smirnov and Shapiro-Wilk tests). The data are presented as medians because of the non-normal distribution. Statistical analysis of the results was performed, and the statistical significance of differences was tested using a Kruskal-Wallis test (*post hoc* Mann-Whitney test). The relationship between two variables was established using Spearman correlation analysis (rho value). The sensitivity and specificity of the variables were analysed using a ROC curve procedure. Differences between groups were considered significant at p < 0.05. Complete statistical analysis of the data was conducted with the statistical software package, SPSS Statistics 17 (Chicago, Illinois, USA).

## Additional Information

**How to cite this article**: Surbatovic, M. *et al.* Cytokine Profile in Severe Gram-Positive and Gram-Negative Abdominal Sepsis. *Sci. Rep.* **5**, 11355; doi: 10.1038/srep11355 (2015).

## Figures and Tables

**Table 1:** Demographic data.

**Total no. of patients**	**165**
Age (average, range)	52.5 (from 19 to 81 yrs)
Sex, n (%)	
male	61 (37%)
female	104 (63%)
Simplified Acute Physiology Score II—SAPS II score, mean ± SD	55.92 ± 8.94
Acute Physiology and Chronic Health Evaluation II—APACHE II score, mean ± SD	22.43 ± 3.82
Sequential Organ Failure Assessment—SOFA score, mean ± SD	7.22 ± 2.23
Length of ICU stay, mean ± SD, range (days)	15 ± 11 (2–175)
Length of hospital stay, mean ± SD, range (days)	53 ± 32 (2–360)
Blood cultures, n (%)	
Gram-positive (GPB)	80 (48.5%)
Gram-negative (GNB)	65 (39.4%)
Polymicrobial (POLY)	20 (12.1%)
Overall hospital mortality	53.30%

**Table 2:** Comparison of Gram-positive and Gram-negative abdominal sepsis according to the outcome.

**Groups**	**Number of patient, (%)**		
	**Non-survivors**	**Survivors**	**Total**
Gram-positive sepsis	35 (43.8)	45 (56.2)	80 (100.0)
Gram- negative sepsis	41 (63.1)	24 (36.9)	65 (100.0)
Total	76 (52.4)	69 (47.6)	145 (100.0)
χ^2^ = 4.624 p = 0.032			

**Table 3:**
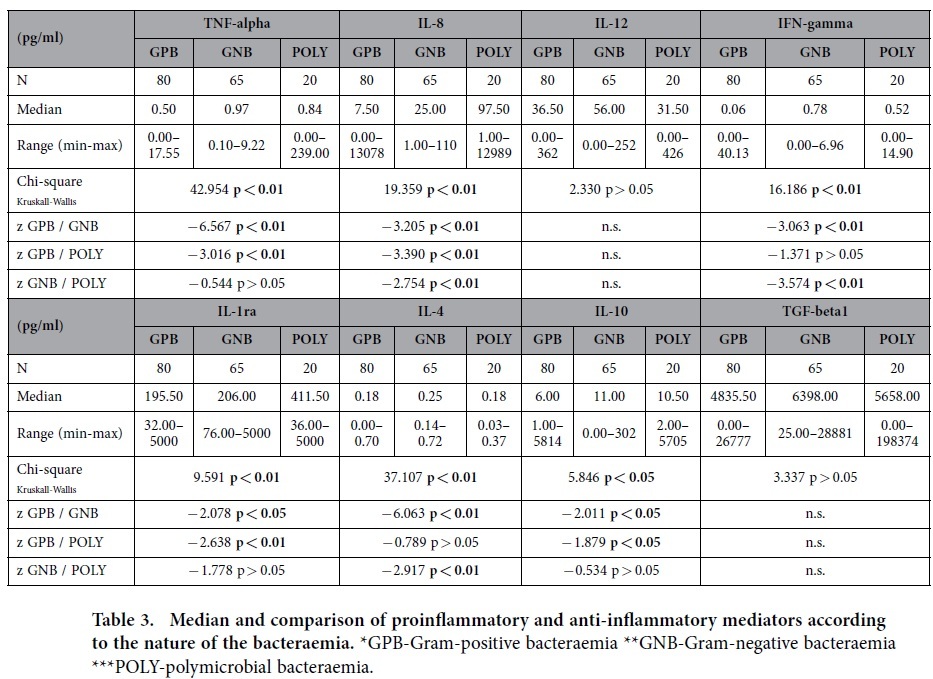
Median and comparison of proinflammatory and anti-inflammatory mediators according to the nature of the bacteraemia.

**Table 4:** Area under the ROC curve for individual cytokine measurements and the presence of Gram-positive and Gram-negative abdominal sepsis.

**AUC/ROC data regarding inflammatory mediators and Gram-positive bacteraemia**						
**Mediator**	**AUC**	**95% CI**	**Sensitivity**	**Specificity**	**Cut-off (pg/mL)**	***p***
TNF-alpha	0.542	0.410–0.674	low	low	N/A	p > 0.05
IL-8	0.719	0.590–0.848	80%	73.3%	<7.50	0.001
IL-12	0.310	0.180–0.439	low	low	N/A	p > 0.05
IFN-gamma	0.489	0.355–0.624	low	low	N/A	p > 0.05
IL-1ra	0.655	0.534–0.775	74.3%	53.3%	<123.50	0.018
IL-4	0.508	0.362–0.653	low	low	N/A	p > 0.05
IL-10	0.786	0.683–0.889	68.6%	68.9%	<6.50	0.000
TGF-beta1	0.327	0.199–0.455	low	low	N/A	p > 0.05
**AUC/ROC data regarding inflammatory mediators and Gram-negative bacteraemia**						
**Mediator**	**AUC**	**95% CI**	**Sensitivity**	**Specificity**	**Cut-off (pg/mL)**	***p***
TNF-alpha	0.912	0.830–0.993	90.2%	87.5%	>0.56	0.000
IL-8	0.999	0.780–1.00	97.6%	100%	>11.50	0.000
IL-12	0.632	0.453–0.810	85.4%	62.5%	>18.50	0.045
IFN-gamma	0.528	0.388–0.669	low	low	N/A	p > 0.05
IL-1ra	0.662	0.530–0.793	58.5%	54.2%	>200.00	0.031
IL-4	0.551	0.410–0.712	low	low	N/A	p > 0.05
IL-10	0.799	0.686–0.911	75.6%	87.5%	>10.50	0.000
TGF-beta1	0.025	0.000–0.067	low	low	N/A	p > 0.05

**Table 5:**
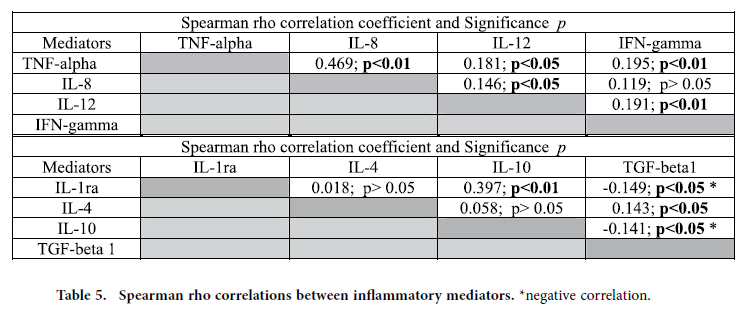
Spearman rho correlations between inflammatory mediators.

**Table 6:**
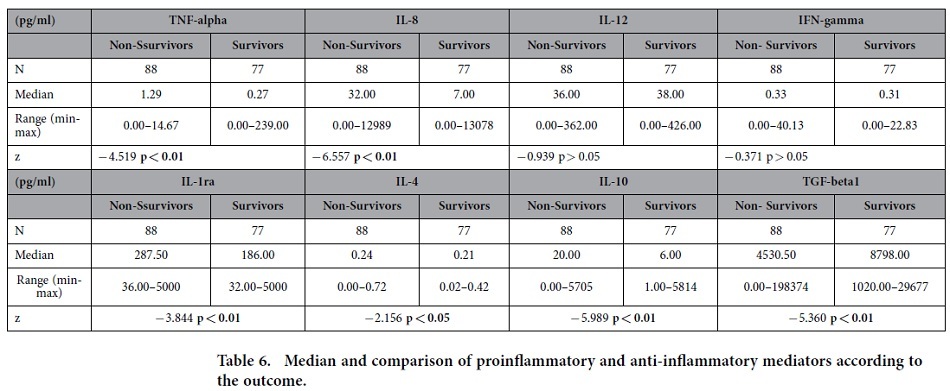
Median and comparison of proinflammatory and anti-inflammatory mediators according to the outcome.

**Table 7:** Area under the ROC curve for individual cytokine measurements and lethal outcome.

**Mediator**	**AUC**	**95% CI**	**Sensitivity**	**Specificity**	**Cut-off (pg/mL)**	***p***
TNF-alpha	0.702	0.618–0.786	67%	76.6%	>0.48	0.000
IL-8	0.796	0.719–0.873	84.1%	80.5%	>11.5	0.000
IL-12	0.458	0.368–0.547	low	low	N/A	p > 0.05
IFN-gamma	0.516	0.428–0.605	low	low	N/A	p > 0.05
IL-1ra	0.674	0.592–0.755	60.2%	54.5%	>200.5	0.000
IL-4	0.597	0.510–0.685	64.8%	53.2%	>0.21	0.031
IL-10	0.770	0.694–0.846	72.7%	80.5%	>9.5	0.000
TGF-beta1	0.258	0.183–0.333	low	low	N/A	p > 0.05
